# Modelling mitochondrial site polymorphisms to infer the number of segregating units and mutation rate

**DOI:** 10.1098/rsbl.2009.0104

**Published:** 2009-03-25

**Authors:** Michael D. Hendy, Michael D. Woodhams, Andrew Dodd

**Affiliations:** 1 Allan Wilson Centre, Massey UniversityPalmerston North 4474, New Zealand; 2 Allan Wilson Centre, Massey UniversityAuckland 0745, New Zealand

**Keywords:** mitochondrial DNA, mutation rate, segregating units, pedigree analysis, Adélie penguins

## Abstract

We present a mathematical model of mitochondrial inheritance evolving under neutral evolution to interpret the heteroplasmies observed at some sites. A comparison of the levels of heteroplasmies transmitted from mother to her offspring allows us to estimate the number *N*_*x*_ of inherited mitochondrial genomes (segregating units). The model demonstrates the necessity of accounting for both the multiplicity of an unknown number *N*_*x*_, and the threshold *θ*, below which heteroplasmy cannot be detected reliably, in order to estimate the mitochondrial mutation rate *μ*_m_ in the maternal line of descent. Our model is applicable to pedigree studies of any eukaryotic species where site heteroplasmies are observed in regions of the mitochondria, provided neutrality can be assumed. The model is illustrated with an analysis of site heteroplasmies in the first hypervariable region of mitochondrial sequence data sampled from Adélie penguin families, providing an estimate *N*_*x*_ and *μ*_m_. This estimate of *μ*_m_ was found to be consistent with earlier estimates from ancient DNA analysis.

## 1. Introduction

It has recently been found that estimates of the human mitochondrial mutation rate from pedigree data are many times higher than those estimated from sequence divergence at putatively neutral sites (e.g. Parsons *et al*. 1997; Howell *et al*. 2003; Santos *et al*. 2005). One possible reason for this is that heteroplasmy (variation among different organelle genomes within the same individual) may be maintained for many generations, after the origin by mutation of a new variant, provided that there is not a very small bottleneck in the number of organelle genomes during transmission from parent to offspring (reviewed by Birky 1991). The persistence of heteroplasmic mutations for many generations after their origin may lead to an inflated contribution to a pedigree-based estimate of mutation rate, if they are treated as mutations that have become fixed within individuals. We propose a method for estimating the mutation rate and size of the transmission bottleneck for maternally transmitted mitochondrial genomes. We illustrate this using the dataset of Millar *et al*. (2008) on the Adélie penguin.

Millar *et al*. (2008) sequenced a segment of 344 base pairs in a fast evolving region of first hypervariable region (a region they argued was not under strong selective pressure) from 1931 Adélie penguins (508 families) at Antarctic nesting sites. This was a follow-up to the study of Lambert *et al*. (2002) of ancient and contemporary samples of the same region of mitochondrial DNA. In the data reported in Millar *et al*. (2008), a low proportion of the sites in individual birds exhibited heteroplasmies, where two bases were called on the electropherogram at that site, each with a signal greater than *θ*=0.23 of the total signal. Signals below the detection threshold *θ*, could not be reliably distinguished from background noise and were not reported there. Fifty-five mothers (out of 508) exhibited site heteroplasmies (above *θ*), with seven having two heteroplasmic sites. Hence, the proportion of sites with an observed heteroplasmy across all mothers was

(1.1) $$\hat{\beta }=\frac{48+2\times 7}{508\times 344}=3.55\times 10^{-4}.$$

In all but three cases, the observed site heteroplasmies persisted above the threshold in both mother and chicks. Site heteroplasmies in a chick were only checked at sites where a heteroplasmy had been observed in its mother.

Following Lambert *et al*. (2002) and Millar *et al*. (2008), we define mutation rate as the rate at which a base substitution is incorporated into all mitochondrial genomes of an individual. Previous studies linking mutation rate with observed site heteroplasmies include two studies (Brandstätter *et al*. 2004; Santos *et al*. 2005) of human populations of up to three generations, where the mutation rate was estimated from assuming that each observed site heteroplasmy represented a transitional substitution. We find most of the substitutions giving rise to an observed site heteroplasmy are subsequently lost, and those that eventually become fixed may persist as an observed site heteroplasmy for many generations and be oversampled.

For our study, we follow the maternal ancestry of an individual, tracking the trajectory of a site substitution introduced into the germ line by some ancestor. At each generation, the population of mitochondrial genomes will pass through a bottleneck when the oocyte segregates a small number of its mother's genomes. Lambert *et al*. (2002) argued that this region of the mt-genome is not under strong selective pressure, so our model assumes random drift among the *N*_*x*_ segregating genomes.

This model should be applicable for any region of the mt-genome assumed neutral under selection for any eukaryote species.

## 2. Material and methods

Our model focuses on an individual site of the mitochondrial genomes in the maternal ancestry of one individual. When a heteroplasmy (comprising two different nucleotides, generically X, Y) is observed at a site, we presume, this is a consequence of a somatic substitution X→Y or Y→X at that site in a maternal ancestor some *g* generations prior, inherited by a daughter, which has persisted for *g* generations.

Consider the maternal ancestry of an individual *A*_0_, with *A*_1_ its mother and *A*_2_ its maternal grandmother, etc. Suppose a nucleotide substitution X→Y has occurred at a site in one genome of a maternal line ancestor *A*_*g*_ (*g*≥1), which is inherited by *A*_*g*−1_. Our model shows that the probability of an additional substitution inherited at that site among *A*_*g*−1_, …, *A*_0_ is less than 10^−2^, so we will neglect this possibility. Suppose this site heteroplasmy persists for (exactly) *k*≥1 generations, inherited by *A*_*g*−1_, …, *A*_*g*−*k*_, but not by *A*_*g*−*k*−1_. If *k*≥*g*, then *A*_0_ will be heteroplasmic at that site, although not necessarily observable. If *k*<*g*, *A*_*g*−*k*−1_, …, *A*_0_ are not heteroplasmic at that site, with their genomes either containing all X or all Y.

We propose a model where each oocyte recruits *N*_*x*_ mitochondrial genomes independently from the population of its mother's genomes. (The number *N*_*x*_ is sometimes referred to as the *number of segregating units*. For the Adélie penguins, we estimated *N*_*x*_ to have a 95% confidence interval (CI) of 25<*N*_*x*_<69.)

The observed levels of most site heteroplasmies from the blood samples of a mother and her chicks closely agree, which reflects a close agreement in the germ line. We will assume that the variation, after accounting for measurement uncertainty, is due to sampling at the recruiting bottleneck and that the proportions in the blood sample estimate the inherited proportions.

If *A*_1_ (the mother of *A*_0_) contains a site heteroplasmy with the nucleotides Y and X appearing in proportions *ϕ* and 1−*ϕ*, then the probability (using a binomial selection with replacement) that *A*_0_ inherits *i* genomes with allele Y and *N*_*x*_−*i* genomes of allele X is

$$\text{Pr}(i|N_{x},\phi )=\left(\begin{array}{c} N_{x}\\ i \end{array}\right)\phi^{i}{\left(1-\phi \right)}^{N_{x}-i}.$$

If *A*_1_ had inherited *j* copies of allele Y and *N*_*x*_−*j* of allele X from her mother *A*_2_, we assume she exhibits the proportions *ϕ*=*j*/*N*_*x*_ and 1−*ϕ*=1−*j*/*N*_*x*_ of the alleles in the genomes available for inheritance. Hence,

(2.1) $$p_{N_{x}}(i,j)=\left(\begin{array}{c} N_{x}\\ i \end{array}\right){\left(\frac{j}{N_{x}}\right)}^{i}{\left(\frac{N_{x}-j}{N_{x}}\right)}^{N_{x}-i},$$

is the probability that *A*_0_ inherits *i* copies of allele Y, and *N*_*x*_−*i* copies of allele X, given her mother had inherited *j* and *N*_*x*_−*j* corresponding copies. Let

(2.2) $$P_{N_{x}}=[p_{N_{x}}(i,j)],$$

be the matrix of these probabilities, where the *N*_*x*_−1 rows and columns are indexed by *i*,*j*∈{1,2, …, *N*_*x*_−1}.

Given that *A*_*g*_ has a somatic mutation in a descendant of one of her *N*_*x*_ founding genomes, the probability that *A*_*g*−1_ inherits more than one mutated genome is very small. Hence, we will assume *A*_*g*−1_ is heteroplasmic at that site, with proportion 1/*N*_*x*_ of its genomes containing Y. If the heteroplasmy is lost *g* generations later, then *A*_0_ has either all X or all Y at that site. Let *h*_X_,_Y_ be the proportion of cases where the heteroplasmy persists and *h*_X_ and *h*_Y_ be the proportions where the mutation is lost or fixed. The neutral model predicts that as *g* increases

$$h_{X,Y}\to 0,\;\;h_{X}\to \frac{N_{x}-1}{N_{x}},\;\;h_{Y}\to \frac{1}{N_{x}}.$$

In a simulation study of 10^7^ heteroplasmic site histories, we followed the introduction of one mutation until the site heteroplasmy was lost, for *N*_*x*_=20 and for *N*_*x*_=40. We found (table 1) for *θ*=0.23 that *h*_X_≈((*N*_*x*_−1)/*N*_*x*_) and *h*_Y_≈1/*N*_*x*_. Table 1 also gives the average numbers of generations that the site heteroplasmies persist, and are observable. We note that over all histories, the average numbers of generations a site heteroplasmy was observable were almost identical for *N*_*x*_=20 and 40, but the average variation in the levels of a heteroplasmy at a site between a mother and her chick differed significantly.

For each *A*_*k*_ in the ancestry, let *n*_*k*_ be the number of its founding genomes with nucleotide Y at that site. We have assumed that *n*_*g*−1_=1. If for *k*<*g*−1, *n*_*k*_=0 or *N*_*x*_, the heteroplasmy is lost. Suppose 1≤*n*_*k*+1_=*j*<*N*_*x*_, then the probability that *n*_*k*_=*i*, (1≤*i*<*N*_*x*_) is

$$\text{Pr}(n_{k}=i|n_{k+1}=j)={\left(P_{N_{x}}\right)}_{i,j}.$$

In lemma 1 of the electronic supplementary material, we show that

$$\text{Pr}(n_{k}=i|n_{g-1}=1)={\left(P_{N_{x}}\right)}_{i,1}^{\left(g-k-1\right)},$$

which is the *i*th entry of the leading column of $P_{N_{x}}^{\left(g-k-1\right)}$. Assuming that the probability that *A*_*g*_ introduces a somatic mutation into the germ line at a selected site is *α*, then summing over all generations *g*≥1, the probability that *A*_0_ has a site heteroplasmy with *n*_0_=*i* (1≤*i*<*N*_*x*_) is

(2.3) $$\text{Pr}(n_{0}=i)=\sum_{g\geq 1}\alpha {\left(P_{N_{x}}\right)}_{i,1}^{\left(g-1\right)}=\alpha {\left(Q_{N_{x}}\right)}_{i,1},$$

where ${\left(Q_{N_{x}}\right)}_{i,1}$ is the first entry in the *i*th row of

$$Q_{N_{x}}=\sum_{g\geq 1}P_{N_{x}}^{\left(g-1\right)}={\left(I-P_{N_{x}}\right)}^{-1}.$$

**Figure 1:**
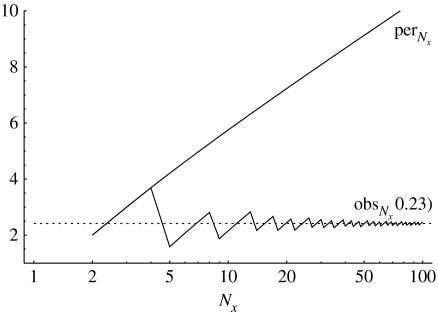
Values of ${\left(Q_{N_{x}}\right)}_{i,1}$ (written as ${\text{per}}_{N_{x}}$), the expected number of generations a site heteroplasmy persists, and of ${\text{obs}}_{N_{x}}(0.23)$, the expected number of generations the site heteroplasmy are observable with threshold *θ*=0.23 (plotted on a logarithmic scale). Note that ${\text{obs}}_{N_{x}}(\theta )$ converges to the constant value 2 ln(*θ*^−1^−1)=2.417 (dotted line), while ${\text{per}}_{N_{x}}$ (solid line) grows logarithmically as *N*_*x*_ increases.

As ${\left(Q_{N_{x}}\right)}_{i,1}$ is the expected number of generations that a new site heteroplasmy persists with *i* copies (figure 1), the expected number of generations a heteroplasmy is observable at that site is

$${\text{obs}}_{N_{x}}=\sum_{\theta \leq i/N_{x}\leq 1-\theta }{\left(Q_{N_{x}}\right)}_{i,1}.$$

Most site heteroplasmies never reach the detection threshold, those that do, usually persist for many more than ${\text{obs}}_{N_{x}}$ generations (table 1).

**Figure 2:**
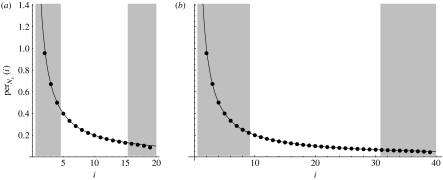
The values of per_20_(*i*), (*a*) for *i*=2, …, 19, and per_40_(*i*), (*b*) for *i*=2, …, 39, are plotted as dots. The solid lines are the curves 2/*i*. The shaded regions are outside the detection threshold for *θ*=0.23 with the observable region (unshaded) between them. We note that in both observed regions the points ${\text{per}}_{N_{x}}(i)$ fit the curve 2/*i* closely.

In figure 2, we plot the values of (*Q*_20_)_*i*,1_ and (*Q*_40_)_*i*,1_, noting that these two distributions are almost identical, and that ${\left(Q_{N_{x}}\right)}_{i,1}$ is closely approximated by 2/*i* within the observed region. (The limit ${\left(Q_{N_{x}}\right)}_{i,1}\to 2/i$ as *N*_*x*_→∞ was noted by Fisher and Wright (Ewens 2004, eqn (1.56)).)

We show in lemma 2 of the electronic supplementary material, that assuming $Q_{N_{x}}(i)\approx (2/i)$, the probability *β* that a site has an observable heteroplasmy is closely approximated by 2*α* ln(*θ*^−1^−1). Assuming neutral evolution, 1/*N*_*x*_ of the substitutions entering the germ line will become fixed in the maternal line of descent, so that the mutation rate can be estimated as

(2.4) $$\mu_{\text{m}}=\alpha /N_{x}t\approx \frac{\beta }{2N_{x}t\;\text{ln}(\theta^{-1}-1)},$$

where *t* is the generation time.

## 3. Results

We have shown that majority of heteroplasmies cannot be observed, leading to undersampling; but that those that do may persist for many generations, leading to oversampling. These two effects do not balance. We have demonstrated that the estimate of the mutation rate from the density of observed heteroplasmic sites is dependent both on the number of segregating units *N*_*x*_ and on the detection threshold *θ*.

For the Adélie penguin data of Millar *et al*. (2008), with *θ*=0.23, *t*=6.46 and $\hat{\beta }=3.55\times 10^{-4}$, we used Bayesian analysis (see ‘Estimating N_x_ from mother–chick comparisons’ of the electronic supplementary material) to obtain a maximum-likelihood estimate of *N*_*x*_=36.5, with 95% CI of 25.0≤*N*_*x*_≤66.9. Using equation (2.4), Millar *et al*. (2008) estimated the median value to be *μ*_m_=0.55 s s^−1^Myr^−1^ (CI 0.29≤*μ*_m_≤0.88), a value not significantly different from the ancient evolutionary rate of *k*=0.86 s s^−1^Myr^−1^ (CI 0.53≤*k*≤1.17) estimated by Lambert *et al*. (2002) for the same region. In their study, Millar *et al*. showed that if they had assumed that each observed heteroplasmy represented a mutation in transition, the estimate of *μ* would have been increased by a factor of nearly 100.

## 4. Discussion

This model has assumed neutral evolution for the regions of mitochondria genome under analysis. Whether specific regions are under selective pressure is outside the scope of this study; however, Rand *et al*. (1994), for example, addressed this issue. Our model is applicable wherever the assumption of neutrality can be assumed for pedigree studies of any species where multiple copies of the mitochondrial genome are inherited, and a sufficient number of heteroplasmies are observed.

Accounting for the effects modelled here may illuminate the apparent discrepancies reported between molecular substitution rates and those estimated from pedigree studies, such as for human studies, e.g. Parsons *et al*. (1997), Howell *et al*. (2003) and Santos *et al*. (2005). As these studies do not report observational thresholds, our model cannot be applied directly to their data.

Improvement in the accuracy of determining the relative expression levels of site heteroplasmies (with $\theta \ll 1/N_{x}$) might lead to a direct estimate of *N*_*x*_. However, it is likely that *N*_*x*_ may vary across the sample, in which case the distribution would not clump around the *i*/*N*_*x*_ values. We have shown (by simulation) that the model is robust against moderate variations in *N*_*x*_, provided we take *N*_*x*_ to be an idealized harmonic mean of the individual values in the sample.

## Figures and Tables

**Table 1:** Site heteroplasmy histories: results from 10^7^ simulations for *N*_*x*_=20 and 40 segregating units. (In more than 99.9% of the histories, the introduced mutation is either lost or fixed within 200 generations, and no site heteroplasmy survived more than 520 generations. Statistics are presented for the cases HX_n_ (X→Y lost and never observed at *θ*=0.23), HX_o_ (lost but observable for at least one generation) and HY (the mutation X→Y is ultimately fixed). We note that the proportions fixed (HY) is close to its expected value of 1/*N*_*x*_. *g*_av_ records the mean number of generations the site heteroplasmy survives, and *g*_av_(obs) records the mean number of generations the site heteroplasmy is in the observable range. Note as *N*_*x*_ is doubled, the *g*_av_(obs) values double, but the proportions are halved so the mean number of generations for which a site heteroplasmy is observable is approximately constant, close to 2.30 in both cases. In the final row, we note the average mean square difference between the mother/chick pairs over all observed heteroplasmic sites differs for *N*_*x*_=20 and *N*_*x*_=40.)

	*N*_*x*_=20			*N*_*x*_=40		
		
category	proportion	*g*_av_	*g*_av_(obs)	proportion	*g*_av_	*g*_av_(obs)
HX_n_	82.47%	3.87	—	91.05%	4.94	—
HX_o_	12.54%	24.26	10.27	6.46%	50.06	19.36
HY	4.99%	37.18	20.75	2.48%	77.54	40.93
all	100.00%	8.09	2.32	100.00%	9.66	2.27
obs. M/C	av. diff.=8.49%			av. diff.=5.98%		

## Supplementary Material

Modelling mitochondrial site heteroplasmies to infer the number of segregating units and mutation rate—Appendix Mathematical proofs of lemmas supporting equation (2.4) and the Bayesian Analysis used to estimate the mitochondrial mutation rate in Adélie penguins
