# Supplementary material for: Modelling mitochondrial site polymorphisms to infer the number of segregating units and mutation rate
Source: Biol Lett. 2009 Mar 25;5(3):397–400. doi: 10.1098/rsbl.2009.0104 (PMC2679937; doi:10.1098/rsbl.2009.0104)
Supplement: Modelling mitochondrial site heteroplasmies to infer the number of segregating units and mutation rate—Appendix — Mathematical proofs of lemmas supporting equation (2.4) and the Bayesian Analysis used to estimate the mitochondrial mutation rate in Adélie penguins [file rsbl20090104s20.pdf]

Electronic Supplementary Material referred to in: Modelling mitochondrial site heteroplasmies to infer the number of segregating units and mutation rate.

Michael D. Hendy, Michael D. Woodhams, Andrew Dodd.

February 18, 2009

Appendix:

Proof of Lemmas

Consider the maternal ancestry $A_k \in \{A_{g-1}, \cdots, A_1, A_0\}$, where for $j > 0$, A_j is the mother of A_{j-1}. Suppose A_{g-1} inherits a mitochondrial site heteroplasmy, with 1 genome having nucleotide `Y` and $N_x - 1$ genomes having nucleotide `X` at that site. Let n_k be the number of founding genomes of A_k with nucleotide `Y` at that site.

Lemma 1

For $1 \leq i < N_x$, the probability that $n_k = i$, given that $n_{g-1} = 1$, is

$$\Pr(n_k = i | n_{g-1} = 1) = (P_{N_x})_{i,1}^{(g-k-1)},$$

the $i-$th entry of the leading column of $P_{N_x}^{(g-k-1)}$.

Proof

We have assumed $n_{g-1} = 1$.

If $n_k = 0$, the mutation is lost, so $n_0 = \cdots = n_k = 0$, and if $n_k = N_x$, the mutation is fixed, so, $n_0 = \cdots = n_k = N_x$. Otherwise, for $k < g-1$, suppose $1 \leq n_{k+1} = j < N_x$ (ie A_{k+1} had inherited j founding genomes with nucleotide `Y`, and $N_x - j$ with nucleotide `X`, at that site), then the probability that $n_k = i$, $(1 \leq i < N_x)$ is

$$\Pr(n_k = i | n_{k+1} = j) = p_{N_x}(i, j).$$

For $1 \leq i < N_x$, let

$$\pi_{N_x}(i, k, g) = \Pr(n_k = i | n_{g-1} = 1)$$

and let

$$\Pi_{N_x}(k,g) = \begin{bmatrix} \pi_{N_x}(1,k,g) \\ \pi_{N_x}(2,k,g) \\ \vdots \\ \pi_{N_x}(N_x-1,k,g) \end{bmatrix}.$$

Then we see for all $k > 1$

$$\begin{aligned} \Pi_{N_x}(k,g) &= P_{N_x}\Pi_{N_x}(k+1,g) \\ &= (P_{N_x})^{(g-k-1)}\Pi_{N_x}(g-1,g) = (P_{N_x})^{(g-k-1)} \begin{bmatrix} 1 \\ 0 \\ \vdots \\ 0 \end{bmatrix}, \end{aligned}$$

which is the leading column of $(P_{N_x})^{(g-k-1)}$. Hence

$$\pi_{N_x}(i,k,g) = (P_{N_x})^{(g-k-1)}_{i,1}.$$

Lemma 2
Assuming the approximation $\mathrm{per}_{N_x}(i) \approx \frac{2}{i}$ *in the observable region, we find the probability that a site has an observable heteroplasmy is approximately*

$$\beta \approx 2\alpha \ln(\theta^{-1} - 1).$$

Proof

$$\begin{aligned} \beta = \alpha\, \mathrm{obs}_{N_x} &= \alpha \sum_{\theta \le i/N_x \le 1-\theta} \mathrm{per}_{N_x}(i) \\ &\approx \alpha \left(\sum_{\theta \le i/N_x \le 1-\theta} \frac{2}{i} \right) \\ &\qquad = 2\alpha(H_{[N_x(1-\theta)]} - H_{[N_x\theta]}), \end{aligned} \tag{1}$$

where H_m is the $m-$th harmonic number. Now H_m converges to, and is well approximated by $\ln(m) + \gamma$ (γ is Euler's constant), so Equation 1 implies

$$\beta \approx 2\alpha\left[\ln(N_x(1-\theta)) - \ln(N_x\theta)\right] = 2\alpha \ln(\theta^{-1} - 1), \tag{2}$$

which is independent of N_x.

Estimating N_x from mother–chick comparisons. A confounding factor in the data of Millar et al (2008) is the measurement error. This increases the perceived difference between the observed levels of site heteroplasmy. We estimated relative site heteroplasmy levels from the relative peak heights of the electropherograms using the methodology reported in Millar et al (2008) for the penguin data, where the accuracy was tested using mixtures of known site heteroplasmies. We found that the standard error in the relative site heteroplasmy measurement was well approximated by the formula

$$\sigma(x) = 0.06(1 - |0.5 - x|),$$

where x is the relative site heteroplasmy level, so $\sigma(x)$ varies from 0.03 to 0.06.

Suppose a chick A_0 inherits i mutant genomes from her mother A_1 who had j copies. As we do not know $N = N_x$, we can only measure their relative levels of site heteroplasmy $r(A_0) = i/N$ and $r(A_1) = j/N$, from blood samples taken from mother and chick. Suppose we have measured the relative levels of site heteroplasmies to be $r_m(A_0)$ and $r_m(A_1)$, with each including an independent random measurement error, which we model as Gaussian. Each data point $(r_m(A_0), r_m(A_1))$, where $r_m(A_1)$ is within the detection threshold

$$\theta \leq r(A_1) \leq 1 - \theta,$$

is included in our analysis. From the analysis of each of these pairs, we estimate N using likelihood maximisation.

An essential step in this process is to be able to derive the likelihood of N,

$$L(N|r(A_0), r(A_1)) = \Pr(r(A_0), r(A_1)|N),$$

for each data point $(r(A_0), r(A_1))$. With $G(\mu, \sigma, x)$ as the Gaussian distribution function, we see

$$\begin{aligned}
\Pr(j|N) &\propto \mathrm{per}_N(j), \qquad (3)\\
\Pr(r(A_1)|j, N) &= G(j/N, \sigma(j/N), r(A_1)),\\
\Pr(r(A_0)|i, N) &= G(i/N, \sigma(i/N), r(A_0)),\\
\Pr(j|r(A_1), N) &\propto \Pr(j|N)\Pr(r(A_1)|j, N),\\
\Pr(i|j, N) &\propto (P_N)_{i,j},\\
\Pr(i|r(A_1), N) &= \sum_j \Pr(i|j, N)\Pr(j|r(A_1), N),\\
\Pr(r(A_0)|r(A_1), N) &= \sum_i \Pr(r(A_0)|i, N)\Pr(i|r(A_1), N),
\end{aligned}$$

and for $\theta \leq r(A_1) \leq 1-\theta$,

$$\begin{aligned} \Pr(r(A_1)|\theta, N) &\propto \sum_j \Pr(r(A_1)|j, N) \Pr(j|N), \\ L(N|r(A_1), r(A_0), \theta) &= \Pr(r(A_1), r(A_0)|\theta, N) \\ &= \Pr(r(A_1)|\theta, N) \Pr(r(A_0)|r(A_1), N). \end{aligned}$$

(Where there are proportionalities, the scaling is calculated by the requirement that the probabilities sum to one. In the case of $\Pr(r(A_1)|\theta, N)$, the normalisation is $\int_\theta^{1-\theta} \Pr(x|\theta, N)\, dx = 1$.)

Equation 3 implicitly makes the assumption that we know which allele is the original, and which is the mutation. In general we may not be able to determine this, but we can make the calculation invariant under the transformation $r(A_1) \to 1-r(A_1)$, $r(A_0) \to 1-r(A_0)$ by the expedient of replacing Equation 3 with

$$\Pr(j|N) \propto \mathrm{per}_N(j) + \mathrm{per}_N(N-j), \tag{4}$$

for $j \leq N/2$, as was done in Millar et al (2008). In that study, the overall likelihood of N is found by multiplying the $L(N|c, m)$ for each of the 123 heteroplasmic site sites in the mother-chick pairs (Figure 1(a)).

By assuming a prior distribution on N, we can convert the calculation into a probability distribution

$$\Pr(N|\mathrm{data}) \propto \Pr(N) L(N|\mathrm{data}), \tag{5}$$

and $\Pr(N|\mathrm{data})$ can be converted to $\Pr(\mu|\mathrm{data})$ via Equation 5. The choice of a flat prior on N implies a non-flat prior on μ, as $\Pr(\mu) \propto 1/\mu$, and vice-versa. We chose the intermediate prior, $\Pr(N) \propto 1/\sqrt{N}$, (equivalently $\Pr(\mu) \propto 1/\sqrt{\mu}$).

In their analysis, Millar et al (2008), posterior probabilities were calculated for each integer N with $10 \leq N \leq 130$, and for each prior, and a smooth curve interpolated through the points (Figure 1(b)). For the intermediate prior, this analysis yielded a maximum probability (mode) value of $N = 36.48$, and the confidence interval $(25.0 - 66.9)$.

Given the posterior probability density function (PDF) $p_N(N)$, we can make a change-of-variable to μ via 5,

$$\begin{aligned} \hat{\mu} &= f(N) = \frac{\hat{\beta}}{aN\, \mathrm{obs}_N}, \\ \bar{p}_\mu(\mu) &= p_N(f^{-1}(\mu))/f'(f^{-1}(\mu)). \end{aligned}$$

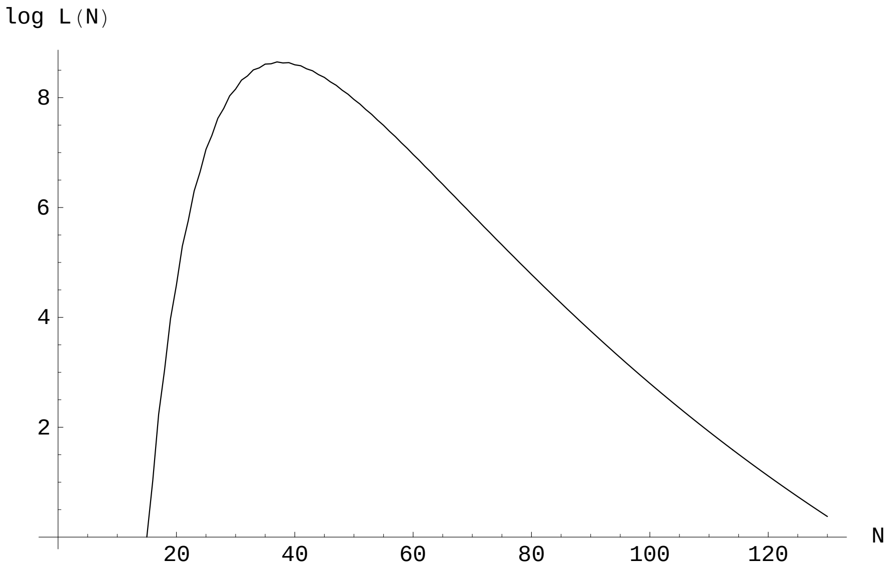

(a)

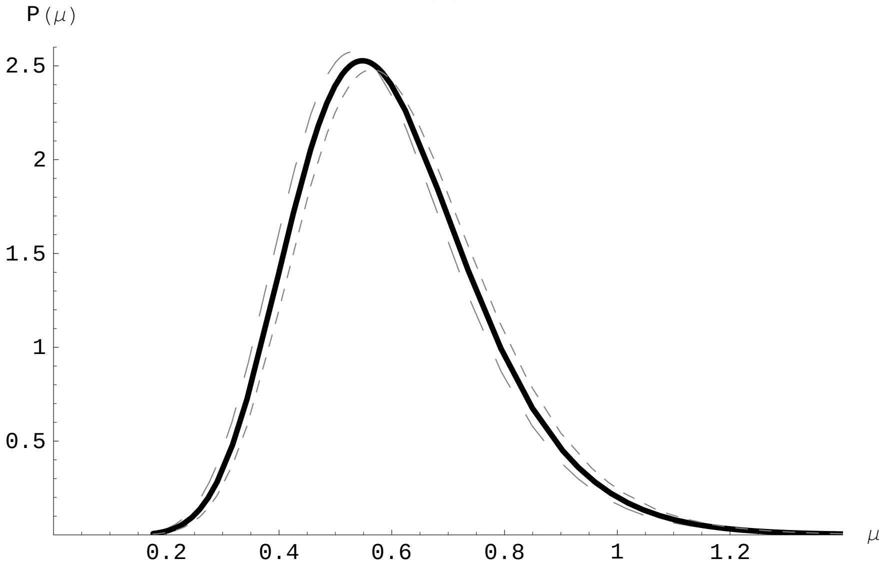

(b)

Figure 1: **(a) Combined log likelihood for N from the 123 mother-chick pair differences. (b) Posterior probability distributions on μ. The dark line is from the intermediate prior $\Pr(\mu) \propto 1/\sqrt{(}\mu)$, the long dashes is for a flat prior on N, and the short dashes is for a flat prior on μ.**

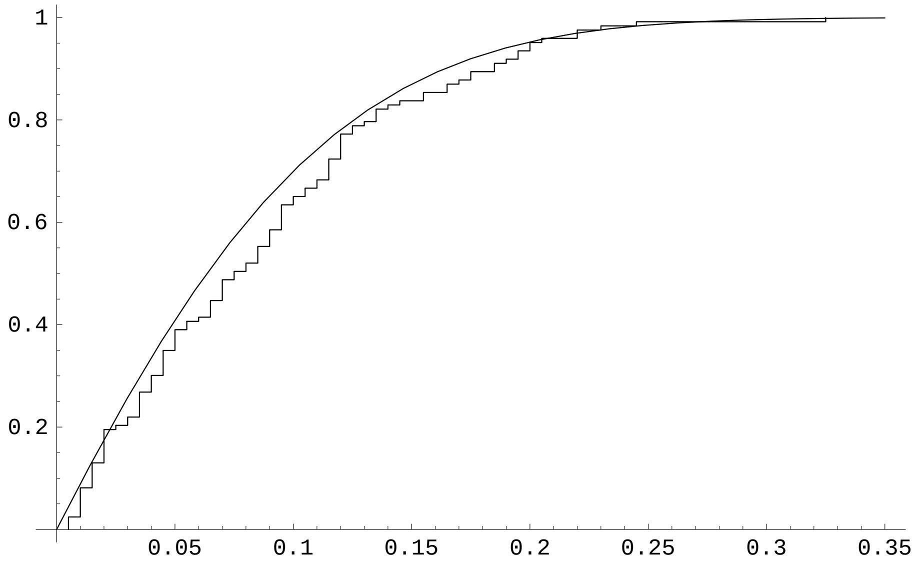

Figure 2: **The cumulative distribution of mother/chick site heteroplasmy differences from Millar et al (2008) plotted against their expected distribution (smooth curve) under the model using $N = 36$.**

Finally, we convolve the PDF $\bar{p}_\mu$ with the uncertainty in $\hat{\beta}$ (as an estimator for β), as the number of heteroplasmic site birds is sampled from a Poisson process. (We do not account for uncertainty in the generation time $t = 6.46$, derived from a table of the ages of nesting mothers in (Millar et al, 2008).)
